# Wessex Head Injury Matrix in Patients with Prolonged Disorders of Consciousness: A Reliability Study

**DOI:** 10.3390/biomedicines12010082

**Published:** 2023-12-28

**Authors:** Maria Daniela Cortese, Francesco Arcuri, Martina Vatrano, Giovanni Pioggia, Antonio Cerasa, Maria Girolama Raso, Paolo Tonin, Francesco Riganello

**Affiliations:** 1 Research in Advanced Neurorehabilitation, S. Anna Institute, Via Siris, 11, 88900 Crotone, Italy; d.cortese@isakr.it (M.D.C.); f.arcuri@isakr.it (F.A.); m.vatrano@isakr.it (M.V.); antonio.cerasa76@gmail.com (A.C.); patonin18@gmail.com (P.T.); 2 Institute for Biomedical Research and Innovation (IRIB), National Research Council of Italy (CNR), 98100 Messina, Italy; giovanni.pioggia@cnr.it

**Keywords:** WHIM, CRS-R, inter-rater reliability, test–retest reliability, prolonged disorders of consciousness

## Abstract

**Introduction:** The Wessex Head Injury Matrix (WHIM) was developed to assess patients with disorders of consciousness (DOC) and was tested in terms of inter-rater reliability (IRR) and test–retest reliability (TRR) in the year 2000. The American Congress of Rehabilitation and Medicine reported that IRR and TRR were unproven. We aim to assess the reliability of the WHIM in prolonged DOC patients (PDOC). **Methods:** A total of 51 PDOC patients (32 unresponsive wakefulness syndrome (UWS/VS) and 19 minimally conscious state (MCS)) who were hosted in a dedicated unit for long-term brain injury care were enrolled. The time from injury ranged from 182 to 3325 days. Two raters administered the Coma Recovery Scale-Revised (CRS-R) and the WHIM to test the IRR and TRR. The TRR was administered two weeks after the first assessment. **Results:** For the CRS-R, the agreement in IRR and TRR was perfect between the two raters. The agreement for the WHIM ranged from substantial to almost perfect for IRR and from fair to substantial for the TRR. **Conclusions:** The WHIM showed a strong IRR when administered by expert raters and strongly correlated with the CRS-R. This study provides further evidence of the psychometric qualities of the WHIM and the importance of its use in PDOC patients.

## 1. Introduction

According to neurological research, consciousness is defined by two key features: (1) wakefulness (i.e., the presence of spontaneous periods with the eyes open); (2) and awareness (i.e., the ability of a subject to respond to internal/external stimuli in an integrated way) [1]. Disorders of consciousness (DOC) define a spectrum of pathologies affecting a person’s ability to interact with the external world. The causes can be traumatic, non-traumatic (such as surgery, infective, vascular, and anoxic), or a combination of both. Two principal conditions characterize DOC patients: the unresponsive wakefulness syndrome/vegetative state (UWS/VS), characterized by spontaneous opening of the eyes and reflexive responses to external stimuli, and the minimally conscious state (MCS) [2], where patients exhibit minimal but discernible signs of non-reflex behaviors which occur reproducibly (but inconsistently) as a response to visual, auditory, tactile, or noxious stimuli [3]. Differentiating the different levels of consciousness is challenging because of the heterogeneous pathologies with diverse etiologies, injuries, and outcomes that characterize DOC.

A recent European Academy of Neurology guideline highlighted the importance of multimodal evaluation in assessing patients with DOC and suggested the implementation of EEG-based techniques and functional neuroimaging [4]. However, despite the utility of neuroimaging and electrophysiology in investigating the content of consciousness in these patients, behavioral assessment remains the gold standard [2].

Many behavioral scales, such as the Coma Recovery Scale-Revised (CRS-R) [5], Wessex Head Injury Matrix (WHIM) [6], Level of Cognitive Functioning Scale [7], and others (see Seel 2010 [8]), have been developed to reduce misdiagnosis errors in patients in UWS/VS and MCS conditions. However, not all scales involve a well-defined administration and sufficiently standardized scoring procedure. A study by the American Congress of Rehabilitation and Medicine (ACRM) [8] reported that scales such as the CRS-R, sensory modality assessment technique [9], and WHIM have well-defined administration and scoring procedures that facilitate consistent use. In contrast, scales such as the Full Outline of UnResponsiveness Score [10] or the Innsbruck Coma Scale [11] have not. The same study suggested that the CRS-R may be used to assess DOC with minor reservations, while scales such as the WHIM may be used to assess DOC with moderate reservations [8].

The CRS-R and WHIM have different approaches to assessing patients. The CRS-R was developed to differentiate UWS/VS and MCS patients. Its scoring is based on the presence of specific behavioral responses to sensory stimuli administered in a standardized manner. It is composed of six sub-scales (e.g., auditory, visual, motor, oromotor/verbal, communication, and arousal) and ordered hierarchically, with the lower items representing reflexive activity and the higher items representing cognitive-mediated behaviors [5].

The WHIM (Supplemental Material, Figure S1) does not directly distinguish UWS/VS and MCS patients but monitors subtle changes. It was developed to identify sequences of recovery processes, encompassing cognitive, social, behavioral, attentive, and communicative aspects [12]. The WHIM is composed of 62 hierarchically organized items. The sequence is organized in a well-defined category of observations regarding the individual’s level of responsiveness and interaction with the environment. The WHIM has a summary score defined by the most advanced behavior (MAB) observed and the total number of different behaviors (TNB) that represent the range of behaviors.

Shiel [6] and Majerus [13] tested the inter-rater reliability (IRR) and test–retest reliability (TRR) of the WHIM on a group of 25 and 5 subjects, respectively. Other studies highlighted the potentiality of WHIM in assessing patients with prolonged DOC (PDOC) [14,15].

WHIM is among the scales recommended for clinical use [3,8]. It has been shown to be promising in assessing and monitoring the recovery of patients with severe head injuries and PDOC, and provides a standardized approach to evaluate cognitive and functional changes over time [16].

However, the detailed work of the ACRM reported that WHIM might be used to assess DOC with moderate reservations and lack evidence of IRR, TRR, internal consistency, and criterion validity [8].

The IRR and TRR results were unproven because they had not been adequately implemented and the methodology was not reported [8]. Consequently, regarding the evidence class of the Task Force classification system for rating risk of bias in IRR methodology, the WHIM was allocated as rank IV (i.e., with a very high risk of bias).

Pistoia and colleagues [17], in a work of translation of the WHIM in the Italian language, studied the IRR and TRR, observing the distribution of Kappa Cohen coefficients for the single items of the scale in a sample of 24 acute severe brain-injured patients, in the acute phase (10 of them had a diagnosis of coma).

Considering the indication of the ACRM, we focused on the IRR and TRR analysis. In our study, the WHIM’s reliability in assessing patients with PDOC was investigated for the first time, comparing the results of the WHIM and CRS-R scores obtained by two expert raters. Considering the substantial stability of the consciousness level of the selected patients, we examined the TRR by comparing assessments with a gap of two weeks between each test.

We expected to find (i) a correlation between the CRS-R total score and WHIM scores, (ii) a good agreement between raters, and, finally, (iii) at least a fair agreement between the scores considering the gap of two weeks between tests.

## 2. Materials and Methods

We enrolled 51 PDOC patients, 32 UWS/VS (11 females, age 55 ± 13; 21 males, age 55 ± 13) and 19 MCS (7 females, age 60 ± 12; 12 males 49 ± 17), in a dedicated unit for long-term acquired brain injury care (LABI) that hosts patients discharged after a minimum of six months of hospitalization in an intensive rehabilitation unit (IRU).

The inclusion criteria for this study were a diagnosis of UWS/VS or MCS based on the CRS-R and more than 180 days from the injury. The exclusion criteria were clinical instability, sepsis, COVID-19 infection, and previous psychiatric disorders. The time from injury was 363 ± 411 days for UWS/VS and 991 ± 1053 days for MCS (Table 1).

Two expert raters with more than 15 years of experience with DOC patients assessed the patients.

Two distinct administration modalities are required for the CRS-R and WHIM. In the first test, designed to identify patients with DOC, the examiner began by rating the highest item—that is, the one that indicated the content of consciousness—and only allocated a score if the behavioral response to the stimulus was observed at least three times. Observations on the individual’s level of responsiveness and involvement with the environment were organized into a clearly defined category in the second test, which was created to identify recovery sequences. All the observed behaviors were marked with “−” if they met the operational definitions; otherwise, they were marked with “+”, ending the assessment after 10 consecutive not-observed behaviors. The last marked item represents the MAB score, and the number of items observed is the TNB score [6].

The patients were nursed before 9:00 a.m. in compliance with the unit rules, and CRS-R and WHIM scales were administered between 9:30 a.m. and 11:30 a.m. to have a higher probability of observing a behavioral response to the stimuli [3,18,19].

For the CRS-R assessment, the examiner evaluated the patients directly according to the guidelines. The scale was administered in the morning on the same day to ensure an accurate score attribution, with at least 30 min elapsing between evaluations to prevent the patient from being overstimulated. The short time lapse between the two raters in the CRS-R administration ensures a low probability of observing a different behavioral response.

**Figure 1:**
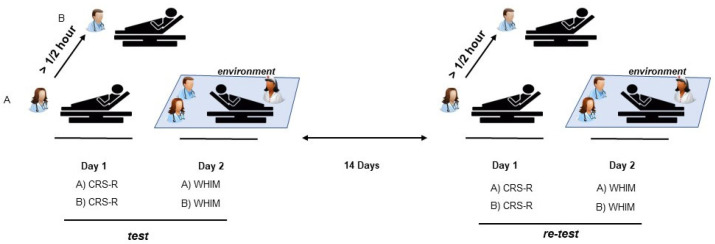
Scheme of CRS-R and WHIM administration. A and B raters assess the patient individually, on the same day, with at least ½ h of distance between them. The patient is assessed contemporaneously by the raters using WHIM on successive day, observing the patient’s interaction with the environment and the nurse. The same procedure is repeated 14 days after.

Conversely, for the CRS-R assessment, to monitor how patients interacted with their surroundings, the WHIM was used by the two raters at the same time on the following day. The behavior of the patient was observed during the nursing interventions, noting the patient’s interactions with the nurse and the raters’ administration of stimuli (i.e., calling the patients by name). The time of administration of the WHIM took approximately 45 min. Considering the slow fluctuation in behavioral response in PDOC patients, the retest was planned two weeks after the first assessment (Figure 1). The IRR was also analyzed in the second week to interpret the data correctly.

The scales were assessed independently without interaction between the two raters.

The Spearman correlation test measured the correlation between the WHIM scores and CRS-R total scores. The level of agreement between raters and consistency across weeks were estimated using the Kappa Cohen test. Kappa values were interpreted as no agreement if k < 0; slight if 0 ≤ k ≤ 0.2; fair if 0.21 ≤ k ≤ 0.4; moderate if 0.41 ≤ k ≤ 0.6; substantial if 0.61 ≤ k ≤ 0.8; and almost perfect if 0.81 ≤ k ≤ 1 [20].

The patients’ relatives and caregivers were informed about the experimental procedure and gave their consent. This study was conducted according to the World Medical Association’s Helsinki Declaration.

## 3. Results

In PDOC patients, the time from injury showed non-normal distributions, with UWS/VS patients having a median of 251 days (skewness = 3.9, kurtosis = 17.4, Kolmogorov–Smirnov *p* < 0.0001) and MCS patients a median of 394 days (skewness = 1.2, kurtosis = 0.1, Kolmogorov–Smirnov *p* < 0.0001).

For both raters, during the test and the retest conditions, the CRS-R correlated positively with the WHIM TNB (Spearman correlation, test raters A and B: Rho = 0.90, *p* = 0.0001; retest raters A and B: Rho = 0.88, *p* = 0.0001) and with the WHIM MAB (Spearman correlation, test rater A: Rho = 0.77, *p* = 0.0001; Spearman correlation, test rater B: Rho = 0.76, *p* = 0.0001; retest raters A and B: Rho = 0.86, *p* = 0.0001) (Table 2).

The two raters showed a perfect IRR for the CRS-R total scores in the test and retest sessions (Cohen’s K = 1 and Cohen’s K = 0.98, respectively). The IRR for the WHIM was almost perfect for the WHIM MAB in the test and retest session (Cohen’s K = 0.96), and almost perfect for WHIM TNB in the test session (Cohen’s K = 0.81) and retest session (Cohen’s K = 0.94).

For rater A, the agreement between the test and retest session was substantial for WHIM MAB (Cohen’s K = 0.62) and fair for WHIM TNB (Cohen’s K = 0.31) and CRS-R (Cohen’s K = 0.28). Similarly, for rater B, the agreement was substantial for WHIM MAB (Cohen’s K = 0.62) and fair for WHIM TNB (Cohen’s K = 0.31) and CRS-R (Cohen’s K = 0.31) (Table 3, Supplemental Material: Tables S1 and S2).

Additionally, we observed IRR and TRR grouping for age (i.e., a1 < 50 yrs; 50 ≤ a2 ≤ 65 yrs; a3 > 65 yrs) and month of hospitalization (m1 ≤ 365 days; m2 > 365 days) (Table 4). For CRS-R, WHIM TNB, and WHIM MAB, the IRR was generally almost perfectly independent of age and month of hospitalization (0.71 ≤ K ≤ 1). Differently, TRR showed a better agreement when considering patients older than 65 yrs (0.55 ≤ K ≤ 67) and a lower agreement when considering patients with an age lower than 50 yrs in WHIM TNB and CRS-R (0.08 ≤ K ≤ 0.30). Differently, WHIM MAB showed a better agreement (K = 0.63). Similarly, for CRS-R and WHIM TNB, better agreement was in TRR when considering patients hospitalized for more than 1 yr (0.34 ≤ K ≤ 0.50), while WHIM MAB had a substantial agreement (k = 0.70) (Table 4).

## 4. Discussion

CRS-R scoring is based on the presence or absence of specific behavioral reactions to standardized sensory stimuli aimed at differentiating and diagnosing UWS/VS, MCS conditions, and the emergence from MCS. It was developed using the Aspen criteria [21]. Conversely, WHIM scoring is based on observed behaviors resulting from the interaction of patients with the environment. It is aimed at identifying recovery processes encompassing cognitive, social, behavioral, attentive, and communicative aspects. Its 62 items are arranged in order of increasing complexity.

In previous studies [6,13], the IRR and TRR were unproven because of the failure to either implement or report an appropriate IRR methodology [8]. The IRR and TRR were tested further on 25 patients in the Shiel study [6] and 5 in the Majerus study [13]. In the Shiel study, the TRR was assessed on the same day, using the first WHIM version consisting of 58 items. In the Majerus study, the IRR and TRR were assessed by observing the behaviors of the patients in the study sample that had been previously recorded on video, using a WHIM version of 66 items. The TRR was performed at least one day after the first assessment. To assess the WHIM reliability, our study used, for the first time, the latest version of the WHIM consisting of 62 items [22].

We enrolled PDOC patients with a time from injury ranging from 182 to 3325 days, with a mean of 991 ± 1053 days for the MCS and 364 ± 411 days for the UWS/VS. The patients were hosted in a dedicated unit for the long-term care of DOC patients. In this unit, the patients continued rehabilitation activity but not in an intensive way. The clinical stability of the patients and the chronic condition prevented us from detecting significant fluctuations in the level of consciousness. The administration of the scales in the morning [19], in terms of the period of high responsiveness [18], increased the probability of observing consistent responses during the interaction with the patient. In addition, the patient’s assessment by CRS-R and WHIM at an interval of 24 h, respectively, ensured coherence between the scale scoring.

Administering the CRS-R on the same days, at different moments of the morning, the two raters were able to assess the patients independently. Conversely, the WHIM was administered at the same time to observe the patient–nurse interaction within the same time frame.

A strong positive correlation was found between the CRS-R and the WHIM MAB and TBN scale scores.

Considering the inter-rater reliability, the two raters showed perfect agreement in the CRS-R assessment and from substantial to almost perfect agreement in the WHIM administration. This confirms that the well-defined administration and scoring procedures of the WHIM facilitate a consistent use [8].

The agreement observed in the test–retest was different: fair for the CRS-R and from fair to moderate for the WHIM scores. These differences were due to the interval of two weeks between the patient’s assessment. However, the slight variation in the score did not change the initial diagnosis, and the agreement between the raters remained strong with a gap of two weeks. Two UWS/VS patients changed the consciousness level in MCS, with an increase in the CRS-R total score from 7 to 10 and from 8 to 11, respectively. For the first patient, the WHIM TNB/MAB scores changed from 9/36 to 10/36 for both raters. For the second patient, the WHIM TNB/MAB scores changed from 16/23 to 17/36 for rater A and from 10/23 to 17/36 for rater B.

Interestingly, in the test–retest, both raters observed changes in 7 patients (14%) (i.e., 1 UWS/VS (3%) and 6 MCS patients (31%)) in the WHIM TNB, while the CRS-R remained constant.

The WHIM approach to assessing patients with PDOC might help in detecting subtle changes that could be characterized by differences in spontaneous behaviors in everyday life (i.e., increasing time with eyes open, or different behavioral responses to environmental stimuli presented spontaneously) [12,13,23].

The serial WHIM evaluations that produced a trajectory of change were correlated with 68% of the variation in PDOC status on discharge from inpatient rehabilitation, reflecting the status at the time of discharge [14].

However, this does not necessarily denote greater overall sensitivity, and careful consideration is needed when comparing these tools due to their differing measurement factors and the inherent complexities in diagnosing disorders of consciousness [16,24].

Additionally, while the WHIM is useful in tracking changes in patient status, it has certain limitations. Specifically, it is less clinically applicable than the CRS-R since it does not directly link assessment outcomes with diagnoses—that is, it does not incorporate specific criteria for diagnosing disorders of consciousness [25].

Turner-Stokes and colleagues [14] proposed a new item order for the WHIM based on their clinical experience, proving the usefulness of this scale as a diagnostic tool in PDOC and suggesting a multicenter study to confirm the results.

Dhamapurkar [15] and colleagues suggested that the WHIM assessment could be used to observe cognitive and physical changes in PDOC patients due to the onset of particular clinical conditions, such as infections, which could impact the response to rehabilitation and recovery.

We found that the WHIM had a strong IRR when administered by expert raters and a strong correlation with the CRS-R. These results highlight the stability of the WHIM and the importance of its use in PDOC patients.

A potential limitation of our study pertains to the methodological difference in the inter-rater reliability assessment for the CRS-R and WHIM, which arose from the inherent nature of these scales. Both raters showed nearly perfect agreement in CRS-R, WHIM TNB, and WHIM MAB for IRR assessment. However, for the CRS-R, two separate presentation and scoring sessions were used, accounting for both scoring variance and patient behavioral variance. In contrast, the WHIM required simultaneous observation by two examiners of the same patient–environment interaction, focusing primarily on scoring variance. This difference implies that achieving a nearly perfect IRR may reflect more strongly on the reliability of the CRS-R compared to the WHIM.

Another limitation of this study was assessing the TRR with a two-week gap. Changes in the level of consciousness could occur within this time, introducing unavoidable differences between the assessments. These differences also depend on age and time from injury. In fact, less variability is observed in TRR in the WHIM MAB for patients older than 65 years and hospitalized for more than one year. Nevertheless, our study demonstrated high inter-rater reliability for both the CRS-R and WHIM during the initial evaluation and after two weeks. This high level of agreement between raters, despite potential slight behavioral changes in patients over time, further supports the reliability of both tools in assessing disorders of consciousness. Moreover, the substantial test–retest reliability of the WHIM MAB underscores the stability and potential reliability of this tool in evaluating patients with PDOC over time.

More studies on the WHIM could address the ACRM study’s limitations and confirm the reliability of the scale in assessing DOC patients.

## Figures and Tables

**Table 1:** Demographic information.

UWS/VS				MCS			
Gender	Age	Time from Injury (Days)	Etiology	Gender	Age	Time from Injury (Days)	Etiology
**female**	69	280	HEM	female	48	214	HEM
	49	200			65	182	
	49	538			71	1591	
	75	309			47	1040	
	56	192			65	183	
	56	219			40	200	
	58	1072			69	2568	TBI
	42	213	ANOX				
	64	182		male	51	295	HEM
	59	280	TBI		76	2143	
	30	250			64	822	
**male**	63	185	HEM		66	241	
	56	297			19	190	TBI
	81	340			70	3325	
	59	968			40	317	
	74	254			44	3012	
	41	277			47	1488	
	62	258	TBI		27	411	
	47	394			47	394	
	29	290					
	45	253					
	49	190					
	42	2320	ANOX		39	194	OTHER
	52	213					
	70	190					
	64	214					
	56	200					
	64	299					
	61	195					
	51	185					
	47	189	OTHER				
	39	194					

UWS/VS: unresponsive wakefulness syndrome/vegetative state; MCS: minimally conscious state; HEM: hemorrhagic; TBI: traumatic brain injury; ANOX: anoxic; OTHER: other etiology.

**Table 2:** Spearman correlation test.

Test			Retest		
**Raters A**			**Raters A**		
	**WHIM TNB**	**WHIM MAB**		**WHIM TNB**	**WHIM MAB**
**CRS-R**	Rho = 0.90; *p* = 0.0001	Rho = 0.77; *p* = 0.0001	**CRS-R**	Rho = 0.88; *p* = 0.0001	Rho = 0.86; *p* = 0.0001
**Raters B**			**Raters B**		
	**WHIM TNB**	**WHIM MAB**		**WHIM TNB**	**WHIM MAB**
**CRS-R**	Rho = 0.90; *p* = 0.0001	Rho = 0.76; *p* = 0.0001	**CRS-R**	Rho = 0.88; *p* = 0.0001	Rho = 0.86; *p* = 0.0001

**Table 3:** IRR and TRR K Cohen Test.

Test (n = 51)			Retest (n = 51)		
**A** vs. **B**					
**WHIM MAB**	**WHIM TNB**	**CRS-R**	**WHIM MAB**	**WHIM TNB**	**CRS-R**
K = 0.96 CI 95% (0.90–1)	K = 0.81 CI 95% (0.70–0.92)	K = 1	K = 0.96 CI 95% (0.90–1)	K = 0.94 CI 95% (0.86–1)	K = 0.98 CI 95% (0.93–1)
**A (n = 51)**			**B (n = 51)**		
**Test** vs. **retest**			**Test** vs. **retest**		
**WHIM MAB**	**WHIM TNB**	**CRS-R**	**WHIM MAB**	**WHIM TNB**	**CRS-R**
K = 0.62 CI 95% (0.47–0.76)	K = 0.31 CI 95% (0.17–0.44)	K = 0.28 CI 95% (0.14–0.43)	K = 0.62 CI 95% (0.47–0.76)	K = 0.31 CI 95% (0.17–0.44)	K = 0.31 CI 95% (0.17–0.44)

**WHIM**: Wessex Head Injury Matrix; **MAB**: Most Advanced Behavior; **TNB**: Total Number of different Behaviors; **CRS-R**: Coma Recovery Scale-Revised. **K**: Cohen test: **k < 0** = no agreement; **0 ≤ k ≤ 0.2** = slight; **0.21 ≤ k ≤ 0.4** = fair; **0.41 ≤ k ≤ 0.6** = moderate; **0.61 ≤ k ≤ 0.8** = substantial; **0.81 ≤ k ≤ 1** = almost perfect. **A and B**: Raters.

**Table 4:** Age and months of hospitalization groups: IRR and TRR K Cohen Test.

	Test			Retest		
	**A** vs. **B**					
	**WHIM MAB**	**WHIM TNB**	**CRS-R**	**WHIM MAB**	**WHIM TNB**	**CRS-R**
a1 (n = 21)	0.94	0.79	1	0.90	0.95	0.94
a2 (n = 20)	0.94	0.71	1	1	0.94	1
a3 (n = 10)	1	1	1	1	0.88	1
m1 (n = 36)	0.97	0.79	1	0.97	0.94	0.97
m2 (n = 15)	0.93	0.85	1	0.92	0.92	1
	**A**			**B**		
	**Test** vs. **retest**			**Test** vs. **retest**		
	**WHIM MAB**	**WHIM TNB**	**CRS-R**	**WHIM MAB**	**WHIM TNB**	**CRS-R**
a1 (n = 21)	0.63	0.30	0.08	0.63	0.29	0.14
a2 (n = 20)	0.53	0.10	0.26	0.53	0.15	0.26
a3 (n = 10)	0.67	0.66	0.65	0.67	0.55	0.65
m1 (n = 36)	0.57	0.22	0.22	0.57	0.27	0.26
m2 (n = 15)	0.70	0.50	0.34	0.70	0.35	0.34

**WHIM**: Wessex Head Injury Matrix; **MAB**: Most Advanced Behavior; **TNB**: Total Number of different Behaviors; **CRS-R**: Coma Recovery Scale-Revised. **K**: Cohen test: **k < 0** = no agreement; **0 ≤ k ≤ 0.2** = slight; **0.21 ≤ k ≤ 0.4** = fair; **0.41 ≤ k ≤ 0.6** = moderate; **0.61 ≤ k ≤ 0.8** = substantial; **0.81 ≤ k ≤1** = almost perfect. **A and B**: Raters Group age: **a1** (less than 50 yrs); **a2** (ranging between 51 and 65 yrs); **a3** (greater than 65 yrs). Group month of hospitalization: **m1** ≤ 365 days; **m2** > 365 days.

## Data Availability

Data are available in Supplementary Materials.

## Supplementary Materials

The following supporting information can be downloaded at https://www.mdpi.com/article/10.3390/biomedicines12010082/s1, Figure S1: Whim; Table S1: test-retest; Table S2: Coma Recovery Scale-R-IRR.
