# Supplementary material for: Wessex Head Injury Matrix in Patients with Prolonged Disorders of Consciousness: A Reliability Study
Source: Biomedicines. 2023 Dec 28;12(1):82. doi: 10.3390/biomedicines12010082 (PMC10813453; doi:10.3390/biomedicines12010082)
Supplement: Supplementary file 1 [file biomedicines-12-00082-s001.zip › biomedicines-2743978-supplementary.pdf]

Wessex Head Injury Matrix in Patients with Prolonged Disorders of Consciousness: A Reliability Study

Maria Daniela Cortese [1], Francesco Arcuri [1], Martina Vatrano [1], Giovanni Pioggia [2], Antonio Cerasa [1,2], Maria Girolama Raso [1], Paolo Tonin [1] and Francesco Riganello [1,*]

**Table S1s**	**test (32 UWS/VS; 19 MCS)**							**re-test (32 UWS/VS; 19MCS)**						
		CRS-R total score		WHIM TNB		WHIM MAB		CRS-R total score		WHIM TNB		WHIM MAB		
	time from injury (days)	A	B	A	B	A	B	A	B	A	B	A	B	aetiology
**UWS/VS**	185	**3**	**3**	2	2	7	7	**5**	**5**	4	4	7	7	HEM
**UWS/VS**	219	**3**	**3**	1	1	7	7	**5**	**5**	3	3	7	7	HEM
**UWS/VS**	340	**4**	**4**	3	3	7	7	**7**	**7**	5	5	14	14	HEM
**UWS/VS**	538	**4**	**4**	5	5	14	14	**5**	**5**	5	5	14	14	HEM
**UWS/VS**	200	**4**	**4**	2	3	17	17	**9**	**9**	10	10	31	31	HEM
**UWS/VS**	280	**4**	**4**	4	4	24	24	**10**	**10**	12	12	32	32	HEM
**UWS/VS**	195	**4**	**4**	3	4	7	7	**5**	**5**	4	4	7	7	ANOX
**UWS/VS**	182	**4**	**4**	4	4	7	7	**5**	**5**	4	4	13	13	ANOX
**UWS/VS**	213	**4**	**4**	2	1	4	3	**5**	**5**	3	3	7	7	ANOX
**UWS/VS**	214	**4**	**4**	4	4	7	7	**5**	**5**	5	5	12	12	ANOX
**UWS/VS**	299	**4**	**4**	4	4	7	7	**5**	**5**	5	6	12	12	ANOX
**UWS/VS**	290	**4**	**4**	4	4	7	7	**4**	**4**	5	4	13	8	TBI
**UWS/VS**	194	**4**	**4**	3	3	7	7	**3**	**3**	4	4	7	7	OTHER
**UWS/VS**	277	**4**	**4**	3	3	7	7	**4**	**5**	3	3	7	7	HEM
**UWS/VS**	200	**5**	**5**	5	5	14	14	**5**	**5**	5	5	14	14	ANOX
**UWS/VS**	192	**5**	**5**	6	7	14	14	**6**	**6**	5	5	14	14	HEM
**UWS/VS**	280	**5**	**5**	3	3	7	7	**7**	**7**	4	4	7	7	TBI
**UWS/VS**	968	**6**	**6**	5	5	12	12	**5**	**5**	6	6	14	14	HEM
**UWS/VS**	309	**6**	**6**	5	5	8	8	**6**	**6**	5	5	14	14	HEM
**UWS/VS**	1072	**6**	**6**	4	4	14	14	**6**	**6**	4	4	14	14	HEM
**UWS/VS**	185	**6**	**6**	8	8	34	34	**8**	**8**	7	7	33	33	ANOX
**UWS/VS**	213	**6**	**6**	6	6	23	23	**8**	**8**	6	6	23	23	ANOX
**UWS/VS**	2320	**6**	**6**	3	3	7	7	**8**	**8**	9	9	23	23	ANOX
**UWS/VS**	250	**6**	**6**	7	7	32	32	**7**	**7**	10	10	32	32	TBI
**UWS/VS**	394	**6**	**6**	8	9	24	24	**7**	**7**	5	5	16	16	TBI
**UWS/VS**	254	**7**	**7**	5	5	15	15	**7**	**7**	5	5	15	15	HEM
**UWS/VS**	297	**7**	**7**	4	4	11	11	**7**	**7**	5	5	11	11	HEM**

**UWS/VS**	190	**7**	**7**	9	9	36	36	**10**	**10**	10	10	36	36	TBI
**UWS/VS**	258	**7**	**7**	7	6	20	20	**7**	**7**	7	7	20	20	TBI
**UWS/VS**	190	**7**	**7**	7	7	14	14	**7**	**7**	7	7	14	14	ANOX
**UWS/VS**	189	**8**	**8**	16	10	23	23	**11**	**11**	17	17	36	36	OTHER
**UWS/VS**	253	**8**	**8**	11	11	24	24	**7**	**7**	15	15	24	24	TBI
**MCS**	182	**9**	**9**	12	13	22	22	**10**	**10**	13	13	22	22	HEM
**MCS**	182	**9**	**9**	13	13	22	22	**9**	**9**	14	14	22	22	HEM**
**MCS**	240	**9**	**9**	10	10	21	21	**11**	**11**	12	12	22	22	HEM
**MCS**	295	**9**	**9**	10	10	28	28	**9**	**9**	11	11	22	22	HEM**
**MCS**	317	**9**	**9**	15	15	24	24	**10**	**10**	15	15	24	24	TBI
**MCS**	394	**9**	**9**	17	17	30	30	**10**	**10**	17	17	30	30	TBI
**MCS**	1488	**9**	**9**	12	12	24	24	**10**	**10**	13	13	24	24	TBI
**MCS**	200	**10**	**10**	16	16	35	35	**10**	**10**	14	14	35	35	HEM**
**MCS**	1040	**10**	**10**	9	9	22	22	**9**	**9**	9	9	22	22	HEM
**MCS**	2143	**10**	**10**	10	10	23	23	**10**	**10**	10	9	23	23	HEM
**MCS**	190	**10**	**10**	16	16	23	23	**12**	**12**	17	17	29	29	TBI
**MCS**	411	**10**	**10**	12	11	24	23	**10**	**10**	12	12	24	23	TBI
**MCS**	2568	**10**	**10**	15	15	22	22	**10**	**10**	14	14	22	22	TBI**
**MCS**	194	**10**	**10**	13	13	28	28	**11**	**11**	15	15	28	28	OTHER
**MCS**	1591	**11**	**11**	25	25	40	40	**13**	**13**	25	25	40	40	HEM
**MCS**	3012	**12**	**12**	19	19	38	38	**12**	**12**	18	18	36	36	TBI**
**MCS**	3325	**12**	**12**	10	10	31	31	**12**	**12**	10	10	31	31	TBI
**MCS**	822	**13**	**13**	20	20	36	36	**13**	**13**	19	19	36	36	HEM**
**MCS**	241	**17**	**17**	16	16	33	33	**17**	**17**	16	16	33	33	HEM

WHIM : Wessex Head Injury Matrix**; MAB:** Most Advanced Behavior**; TNB**: Total Number of different Behaviors**; CRS-R:** Coma Recovery Scale-Revised**. UWS/VS**: vegetative state/unresponsive wakefulness syndrome; **MCS**: minimally conscious state**; HEM:** hemorrhagic; **TBI:** traumatic brain injury**; ANOX:** anoxic**; OTHER:** other aetiology: **A & B**: Raters.
****** both raters observe changes in the WHIM TNB but no in the CRS-R total score

Table S2: Coma Recovery Scale-R - IRR														
	**test (A & B) (n=51)**							**re-test (A & B) (n=51)**						
**patients**	uditive	visual	motor	oro-verbal	communication	arousal	**total score**	uditive	visual	motor	oro-verbal	communication	arousal	**total score**
**1**	0	0	2	1	0	0	**3**	1	0	1	1	0	2	**5**
**2**	1	0	1	1	0	0	**3**	1	0	2	1	0	1	**5**
**3**	0	0	2	1	0	1	**4**	2	0	2	1	0	2	**7**
**4**	1	0	1	1	0	1	**4**	1	0	1	1	0	2	**5**
**5**	1	0	1	1	0	1	**4**	3	2	1	1	0	2	**9**
**6**	1	0	1	1	0	1	**4**	2	3	2	1	0	2	**10**
**7**	1	0	1	1	0	1	**4**	1	0	1	1	0	2	**5**
**8**	1	0	2	0	0	1	**4**	1	0	2	1	0	1	**5**
**9**	1	0	2	1	0	0	**4**	1	0	2	1	0	1	**5**
**10**	1	0	1	1	0	1	**4**	1	1	1	1	0	1	**5**
**11**	1	0	1	1	0	1	**4**	1	1	1	1	0	1	**5**
**12**	1	0	1	0	0	2	**4**	1	0	1	0	0	2	**4**
**13**	1	0	1	1	0	1	**4**	1	0	1	1	0	0	**3**
**14 A**	1	0	2	1	0	1	**5**	1	0	1	1	0	1	**4**
**14 B**	1	1	1	0	0	2	**5**	1	1	1	0	0	2	**5**
**15**	1	0	2	1	0	1	**5**	1	0	2	1	0	1	**5**
**16**	1	0	2	1	0	1	**5**	1	0	2	1	0	2	**6**
**17**	1	1	2	0	0	1	**5**	1	1	2	1	0	2	**7**
**18**	1	0	2	1	0	2	**6**	1	0	2	1	0	1	**5**
**19**	1	0	2	1	0	2	**6**	1	0	2	1	0	2	**6**
**20**	1	0	2	1	0	2	**6**	1	0	2	1	0	2	**6**
**21**	2	1	1	1	0	1	**6**	2	1	1	2	0	2	**8**
**22**	1	1	2	1	0	1	**6**	2	1	2	1	0	2	**8**
**23**	1	1	2	1	0	1	**6**	2	1	2	1	0	2	**8**
**24**	1	0	2	1	0	2	**6**	2	0	2	2	0	1	**7**
**25**	1	0	1	2	0	2	**6**	2	1	1	1	0	2	**7**
**26**	2	1	1	1	0	2	**7**	2	1	1	1	0	2	**7**
**27**	2	1	2	1	0	1	**7**	2	1	2	1	0	1	**7**
**28**	2	0	2	1	0	2	**7**	3	1	2	2	0	2	**10**
**29**	2	1	1	1	0	2	**7**	2	1	1	1	0	2	**7**
**30**	2	0	2	1	0	2	**7**	2	0	2	1	0	2	**7**
**31**	1	1	2	2	0	2	**8**	3	3	1	2	0	2	**11**
**32**	2	1	2	1	0	2	**8**	2	1	2	1	0	1	**7**
**33**	2	3	2	1	0	1	**9**	2	3	2	1	0	2	**10**
**34**	2	1	2	2	0	2	**9**	2	1	2	2	0	2	**9**

**35**	2	3	2	1	0	1	**9**	2	3	4	1	0	1	**11**
**36**	2	2	2	1	0	2	**9**	2	3	1	1	0	2	**9**
**37**	2	2	2	1	0	2	**9**	2	3	2	1	0	2	**10**
**38**	2	3	1	1	0	2	**9**	2	3	1	2	0	2	**10**
**39**	2	3	1	1	0	2	**9**	2	3	2	1	0	2	**10**
**40**	2	3	2	1	0	2	**10**	2	3	2	1	0	2	**10**
**41**	4	0	3	1	0	2	**10**	3	0	3	1	0	2	**9**
**42**	2	3	2	1	0	2	**10**	2	3	2	1	0	2	**10**
**43**	2	3	2	1	0	2	**10**	3	3	2	2	0	2	**12**
**44**	2	3	2	1	0	2	**10**	2	3	2	1	0	2	**10**
**45**	2	3	2	1	0	2	**10**	2	3	2	1	0	2	**10**
**46**	3	3	1	1	0	2	**10**	3	3	2	1	0	2	**11**
**47**	2	3	3	2	0	1	**11**	2	3	4	2	0	2	**13**
**48**	0	3	5	2	0	2	**12**	0	3	5	2	0	2	**12**
**49**	2	2	5	1	0	2	**12**	2	2	5	1	0	2	**12**
**50**	2	3	4	2	0	2	**13**	2	3	4	2	0	2	**13**
**51**	4	5	5	1	0	2	**17**	4	5	5	1	0	2	**17**
	**A & B:** Raters.													

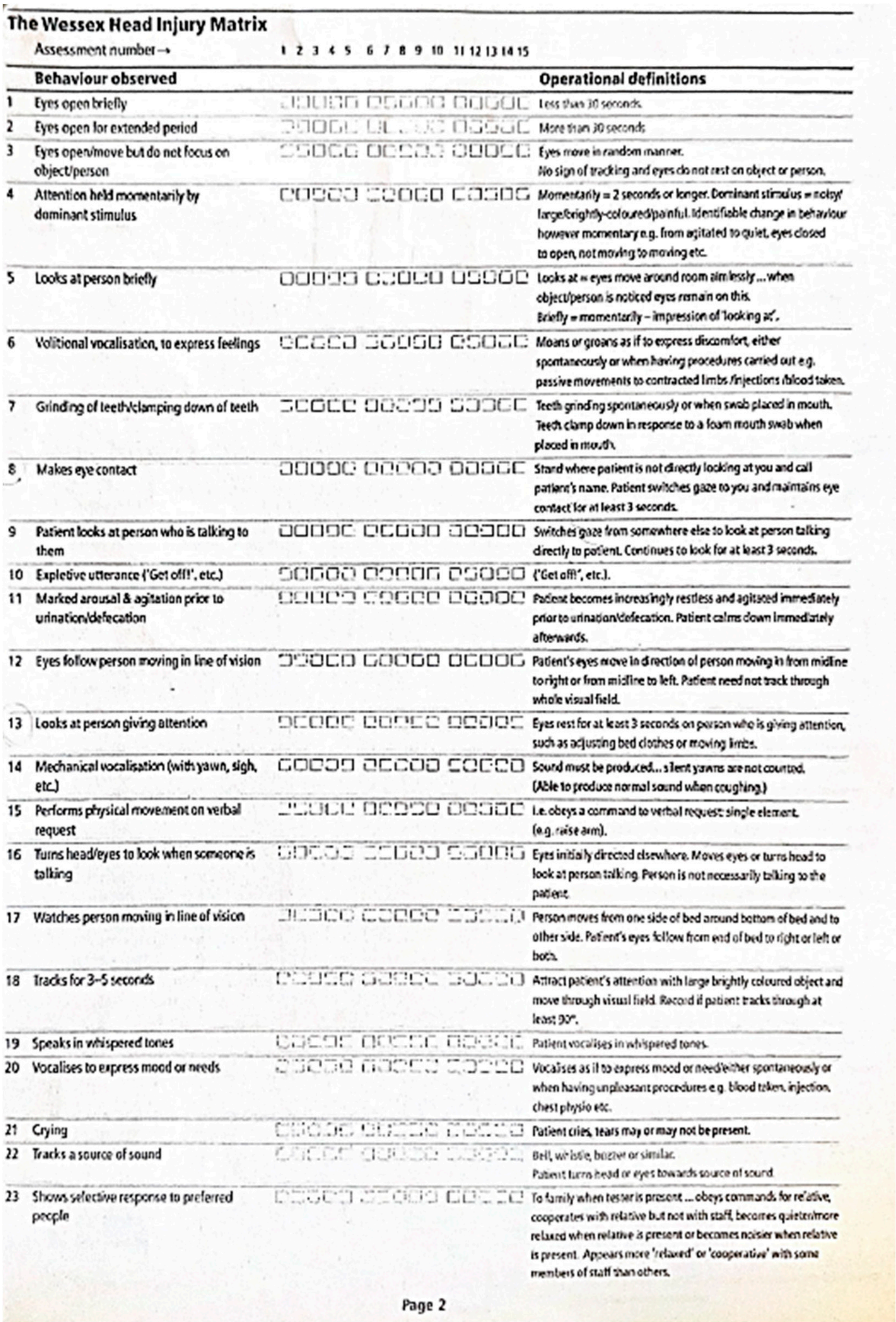

The Wessex Head Injury Matrix

Assessment number → 1 2 3 4 5 6 7 8 9 10 11 12 13 14 15

	Behaviour observed	Assessment (1–15)	Operational definitions
1	Eyes open briefly		Less than 30 seconds.
2	Eyes open for extended period		More than 30 seconds
3	Eyes open/move but do not focus on object/person		Eyes move in random manner. No sign of tracking and eyes do not rest on object or person.
4	Attention held momentarily by dominant stimulus		Momentarily = 2 seconds or longer. Dominant stimulus = noisy/large/brightly-coloured/painful. Identifiable change in behaviour however momentary e.g. from agitated to quiet, eyes closed to open, not moving to moving etc.
5	Looks at person briefly		Looks at = eyes move around room aimlessly ... when object/person is noticed eyes remain on this. Briefly = momentarily – impression of 'looking at'.
6	Volitional vocalisation, to express feelings		Moans or groans as if to express discomfort, either spontaneously or when having procedures carried out e.g. passive movements to contracted limbs /injections /blood taken.
7	Grinding of teeth/clamping down of teeth		Teeth grinding spontaneously or when swab placed in mouth. Teeth clamp down in response to a foam mouth swab when placed in mouth.
8	Makes eye contact		Stand where patient is not directly looking at you and call patient's name. Patient switches gaze to you and maintains eye contact for at least 3 seconds.
9	Patient looks at person who is talking to them		Switches gaze from somewhere else to look at person talking directly to patient. Continues to look for at least 3 seconds.
10	Expletive utterance ('Get off!', etc.)		('Get off!', etc.).
11	Marked arousal & agitation prior to urination/defecation		Patient becomes increasingly restless and agitated immediately prior to urination/defecation. Patient calms down immediately afterwards.
12	Eyes follow person moving in line of vision		Patient's eyes move in direction of person moving in from midline to right or from midline to left. Patient need not track through whole visual field.
13	Looks at person giving attention		Eyes rest for at least 3 seconds on person who is giving attention, such as adjusting bed clothes or moving limbs.
14	Mechanical vocalisation (with yawn, sigh, etc.)		Sound must be produced... silent yawns are not counted. (Able to produce normal sound when coughing.)
15	Performs physical movement on verbal request		I.e. obeys a command to verbal request: single element. (e.g. raise arm).
16	Turns head/eyes to look when someone is talking		Eyes initially directed elsewhere. Moves eyes or turns head to look at person talking. Person is not necessarily talking to the patient.
17	Watches person moving in line of vision		Person moves from one side of bed around bottom of bed and to other side. Patient's eyes follow from end of bed to right or left or both.
18	Tracks for 3–5 seconds		Attract patient's attention with large brightly coloured object and move through visual field. Record if patient tracks through at least 90°.
19	Speaks in whispered tones		Patient vocalises in whispered tones.
20	Vocalises to express mood or needs		Vocalises as if to express mood or need either spontaneously or when having unpleasant procedures e.g. blood taken, injection, chest physio etc.
21	Crying		Patient cries, tears may or may not be present.
22	Tracks a source of sound		Bell, whistle, buzzer or similar. Patient turns head or eyes towards source of sound.
23	Shows selective response to preferred people		To family when tester is present ... obeys commands for relative, cooperates with relative but not with staff, becomes quieter/more relaxed when relative is present or becomes noisier when relative is present. Appears more 'relaxed' or 'cooperative' with some members of staff than others.

Page 2

Figure S1: image of the first page of the WHIM (English version). The WHIM scale does not exist in the free version. The French free version is available at https://www.uslegalforms.com/form-library/103332-wessex-head-injury-matrix-pdf.
